# Mycorrhizal type of woody plants influences understory species richness in British broadleaved woodlands

**DOI:** 10.1111/nph.18274

**Published:** 2022-06-15

**Authors:** Petra Guy, Richard Sibly, Simon M. Smart, Mark Tibbett, Brian J. Pickles

**Affiliations:** ^1^ School of Biological Sciences University of Reading, Health and Life Sciences Building Whiteknights Reading RG6 6EX UK; ^2^ School of Agriculture, Policy, and Development University of Reading Whiteknights Reading RG6 6BZ UK; ^3^ UK Centre for Ecology & Hydrology Library Avenue, Bailrigg Lancaster LA1 4AP UK

**Keywords:** arbuscular mycorrhiza, Bunce survey, ectomycorrhiza, forest, herbaceous, mycorrhizal type, species richness, woodland

## Abstract

- Mature temperate woodlands are commonly dominated by ectomycorrhizal trees, whereas understory plants predominantly form arbuscular mycorrhizal associations. Due to differences in plant–fungus compatibility between canopy and ground layer vegetation the ‘mycorrhizal mediation hypothesis’ predicts that herbaceous plant establishment may be limited by a lack of suitable mycorrhizal fungal inoculum.
- We examined plant species data for 103 woodlands across Great Britain recorded in 1971 and in 2000 to test whether herbaceous plant species richness was related to the proportion of arbuscular mycorrhizal woody plants. We compared the effect of mycorrhizal type with other important drivers of woodland plant species richness.
- We found a positive effect of the relative abundance of arbuscular mycorrhizal woody plants on herbaceous plant species richness. The size of the observed effect was smaller than that of pH. Moreover, the effect persisted over time, despite many woodlands undergoing marked successional change and increased understorey shading.
- This work supports the mycorrhizal mediation hypothesis in British woodlands and suggests that increased abundance of arbuscular mycorrhizal woody plants is associated with greater understory plant species richness.

## Introduction

Temperate forests and woodlands are significant repositories of biodiversity, which is currently in decline due to human activity and climate change. Within woodlands, a greater diversity of tree species has been shown to buffer the negative effects of drought (Gazol & Camarero, 2016; Aussenac *et al*., 2019) and increase tree productivity (Fichtner *et al*., 2017), whilst plant diversity more broadly is an essential component of ecosystem health, productivity, and resilience to multiple types of disturbance (Hector *et al*., 1999; Loreau *et al*., 2001; Loreau & Hector, 2001; van der Plas, 2019). Therefore a major goal in ecology is to understand the mechanisms that determine the diversity and composition of plant communities and their stability over time.

Plant community structure and diversity are linked by complex plant–soil feedback (PSF) mechanisms (Bever *et al*., 1997; van der Heijden *et al*., 1998; van der Heijden & Horton, 2009) that influence both above and belowground assemblages of organisms (Hartnett & Wilson, 2002; Wardle *et al*., 2004; Johnson *et al*., 2005; van der Putten *et al*., 2013; Kardol *et al*., 2015; Ke *et al*., 2015; Tedersoo *et al*., 2020). For example, tree species that acquire pathogenic root fungi at a greater rate than mutualistic fungi are more likely to suffer from negative density dependence (Chen *et al*., 2019). An important trait that influences PSFs is the mycorrhizal type of plants (Moora, 2014).

Mycorrhizas are an ancient association between plants and mycorrhizal fungi (Lutzoni *et al*., 2018; Strullu‐Derrien *et al*., 2018) in which host plants provide the fungi with photosynthate in exchange for access to soil nutrients and other services (van der Heijden *et al*., 2015). An increasing number of different mycorrhizal types are now recognised (Kariman *et al*., 2018), but temperate trees and other woody plants are typically either ectomycorrhizal (EM) hosts (colonised by EM fungi), or arbuscular mycorrhizal (AM) hosts (colonised by AM fungi), although some plant species can associate with both (dual‐mycorrhizal; Teste *et al*., 2019). Many of these fungi can colonise multiple individual plants, forming a common mycorrhizal network (CMN) (Leake *et al*., 2004; Simard & Durall, 2004; Simard *et al*., 2012) capable of transferring nutrients and defence signals, and potentially providing other benefits such as drought tolerance (Finlay & Read, 1986; Gorzelak *et al*., 2015; Gehring *et al*., 2017; Pickles & Simard, 2017). These CMNs mediate plant community structure (Booth, 2004; McGuire, 2007; Simard, 2009) by increasing seedling survival through access to compatible mycelia growing on adjacent conspecific or heterospecific host trees (Simard *et al*., 1997, 2012; Selosse *et al*., 2006; McGuire, 2007; van der Heijden & Horton, 2009; Liang *et al*., 2020).

A growing body of evidence indicates that mycorrhizal associations and CMNs tend to produce different responses in their hosts, with EM associations commonly generating positive to neutral PSFs and AM associations neutral to negative PSFs (van der Heijden & Horton, 2009; Bennett *et al*., 2017; Teste *et al*., 2017; Kadowaki *et al*., 2018). Haskins & Gehring (2005) demonstrated that pinyon pine (*Pinus edulis*) seedlings, an EM host, were less colonised by EM fungi when growing near AM type trees. In other words, the sources of EM fungal inoculum were limited in soil in which AM type hosts were dominant. Similarly, the successful colonisation of AM‐dominated grasslands (Thiet & Boerner, 2007) and heath (Collier & Bidartondo, 2009) by EM type seedlings may be limited by low levels of EM fungal inoculum. Weber *et al*. (2005) demonstrated that AM type trees (western redcedar; *Thuja plicata*) could be excluded from forest areas dominated by EM type trees due to a lack of AM fungal inoculum. Kovacic *et al*. (1984) found a lack of AM fungal inoculum under live EM type pines (ponderosa pine; *Pinus ponderosa*) compared with dead pines and observed a higher abundance of AM type understorey plants beneath dead rather than live pines. Similarly, Barni & Siniscalco (2000) found that AM fungal inoculum was reduced in sites that had succeeded to predominantly EM type trees. Notably, they found that AM fungal inoculum was still high in the early stages of succession when AM type trees were abundant. Therefore the establishment of plants can be influenced by the supply of compatible AM or EM fungal inoculum, with the potential to affect range dynamics of trees over sufficient timescales (Pither *et al*., 2018).

The ‘mycorrhizal mediation hypothesis’ proposed by Veresoglou *et al*. (2017) suggests that AM‐associated woody plants will facilitate the establishment, and therefore potentially increase the species richness, of AM‐associated herbaceous plants. The relationship between AM trees and herbaceous plant species richness was first explored over 30 yr ago. Newman & Reddell (1988) found a strong positive correlation between the relative abundance of AM trees and the species richness of herbaceous plants in a study of plant communities in the Great Smoky Mountains, Tennessee (USA). More recently, Veresoglou *et al*. (2017) speculated that this positive relationship was due to mycorrhizal mediation through inoculum supply. Using data from 77 mixed broadleaf woodlands in north‐western Germany, they found that the richness and abundance of herbaceous plants was positively correlated with the abundance of AM trees and woody shrubs. In a subset of the same woodlands, increasing AM tree cover (%) was not found to be related to the diversity of AMF soil communities (Grünfeld *et al*., 2021), but did appear to influence the colonisation rate of understory AM plant species (Grünfeld *et al*., 2019). This suggests that mycorrhizal mediation between trees and herbaceous plants may be an important driver of herbaceous plant species richness in woodlands.

Most broadleaved woodlands in Great Britain are dominated by EM rather than AM hosts (please refer to Supporting Information Fig. S1). According to the National Forest Inventory (NFI), only a quarter of broadleaved tree cover in 2011 was provided by AM hosts (National Forest Inventory, 2012). Much of this (44%) was formed by ash (*Fraxinus excelsior*), which is currently in decline due to the invasive emerald ash borer (*Agrilus planipennis*) and *Chalara* ash dieback (*Hymenoscyphus fraxinus*). Therefore, if herbaceous plant species richness is related to AM tree cover, this may have important consequences for woodland ecology and management in Britain.

Here we provide the first comprehensive examination of the mycorrhizal mediation hypothesis in British woodlands using the Bunce survey (Wood *et al*., 2015). The Bunce survey has so far taken place twice, in 1971 and again in 2000. The data set has been thoroughly reviewed elsewhere (Kirby *et al*., 2005; Smart *et al*., 2014) and much is already known about the change in British woodlands over the past 70 yr (Hopkins & Kirby, 2007; Keith *et al*., 2009). For example, a lack of management has tended to change the structure of woodland into more mature high forest with an increase in tree basal area, a reduction in the number of trees with small stems, and a homogenisation of plant species. In other words, a smaller number of shade‐loving species has increased, and a much larger number of light‐loving species has been lost, with increases in understory trees such as holly (*Ilex aquifolium*) that shades out the understory and can lead to a reduction in diversity. A noticeable exception to this trend was the 1987 storm in the southeast of the UK, which introduced open areas and resulted in increased herbaceous plant species richness (Smart *et al*., 2014). However, overall, understorey species richness decreased between the surveys. Soil pH has also tended to increase between the surveys in line with national trends due to reduced sulphur deposition (Kirk *et al*., 2010). No changes were found in mean soil organic matter, although some sites saw significant increases and fewer plots showed low levels of soil organic matter.

In general, climatic gradients are known to influence plant richness, with a general trend towards increased species richness in the south of the UK driven by energy‐related variables (Albuquerque *et al*., 2011), although these are likely to be modified by local topographic effects (O'Brien, 2000). Whilst edaphic data are part of the Bunce survey and are highly precise to the plots at 200 m^2^ resolution, climate data would be at a much lower resolution of 5 km grid squares and would not therefore be able to explain any of the within‐site, between‐plot variation in the response, possibly leading to a fatally underpowered analysis. Moreover, soil pH and carbon content integrated many distal effects including climate, topography, elevation and pollutant deposition. Therefore, whilst climate would be a coarse estimate that may be the same for several sites, edaphic variables are precisely aligned with the plant data.

We therefore asked whether the abundance of AM trees and shrubs influenced herbaceous species richness, and whether this effect was detectable over the 29 yr between surveys and across a uniquely large‐scale but fine‐resolution sample of both less shaded and more shaded, mature woodlands. Additionally, we examined the additive and interactive effects of shading, soil organic matter, and soil pH along with the relative abundance of AM trees and shrubs to compare the effect size of the latter to these other important predictors. Our primary aim was to determine whether the relative abundance of AM trees and shrubs in British woodlands had a positive effect on herbaceous plant species richness, using long‐term, large‐scale monitoring data gathered across Great Britain in 1971 and again in 2001. If true, this would provide an important and independent confirmation of previous work on the mycorrhizal mediation hypothesis (Veresoglou *et al*., 2017; Grünfeld *et al*., 2019). Furthermore, our approach would enable a novel exploration of the strength of any mycorrhizal mediation between trees and herbaceous plants in woodlands as a driver of herbaceous plant species richness, relative to other important factors, and whether any such effect persists over time.

## Materials and Methods

### Sources of data

We used the Bunce survey (Wood *et al*., 2015), which recorded all plant species in 16 randomly placed square permanent 200 m^2^ plots in each of 103 broadleaved seminatural woodlands across Great Britain. The Bunce survey is the only survey of its type in the UK, incorporating long‐term monitoring of multiple woodlands across England, Scotland and Wales. The survey includes both biotic and abiotic data for 103 woodlands, originally selected as being a representative subset of over 2000 sites and are therefore considered to be characteristic of native British woodlands. The herbaceous plant richness comes from the recording of ground cover, which lists all plant species and seedlings of trees and shrubs (defined as individuals below 25 cm in height). Tree species in each plot are recorded separately with diameter at breast height (DBH) and number of stems in each DBH class. Additionally, soil organic matter content (SOM) and soil pH (pH) were measured from a 5 × 15 cm soil sample removed from the centre of each plot. The assignment of mycorrhizal type of the trees and woody shrubs was made after thorough scrutiny of sources cited in available trait databases (Akhmetzhanova *et al*., 2012; Soudzilovskaia *et al*., 2020) together with additional sources where data were scarce or lacking for British species (please refer to Methods S1). The mycorrhizal types are summarised in Table S1.

### Statistical analysis

The species richness (α‐diversity) for the ground flora was calculated for each 200 m^2^ plot. The total woody canopy cover was calculated as the sum over the DBH classes multiplied by the number of stems in that class. This value was used as a proxy for shading (shading: cm). The subset of AM type trees and shrubs was extracted and the AM overstory cover was calculated. The relative abundance of AM type trees and shrubs (RelAm: dimensionless ratio) was then the AM cover divided by the total cover. To estimate the inoculum potential of each plot, we use the correlation between shoot and root biomass. In a meta‐analysis of over 786 studies a positive linear correlation was found between shoot and root biomass in woodlands (Mokany *et al*., 2006). As most fine roots will be colonised by mycorrhizal fungi, a larger tree implies a larger fine root mass and a higher fungal colonisation. Therefore a larger tree has greater inoculum potential, that is, it is more likely to have more fungal material to produce propagules, whether those propagules are mycelia or spore‐containing bodies. In addition, larger trees are generally expected to produce more carbon through photosynthesis and will be more capable of supporting larger mycorrhizal fungal communities. Therefore, based on these aboveground–belowground links, we considered that the aboveground measure of DBH × stem count was a reasonable way of estimating the belowground contribution of AM type trees to AM fungal inoculum potential. One large tree may have the same inoculum potential as several smaller shrubs, but will also increase shading and therefore may have a negative impact on plant richness, therefore our inclusion of the shading term. Soil pH (pH: negative log of H^+^ activity) and SOM (% dry matter lost on ignition) were also extracted from the data.

To account for the nested structure of the data of plots within sites, mixed effects models were used (Gelman & Hill, 2007; Zuur *et al*., 2009; Schielzeth & Nakagawa, 2013). The lme4 package in R (Bates *et al*., 2015) was used for generalised linear mixed effects model (GLMM) analysis. We did not seek here to create a model that incorporated all known effects as prediction of woodland responses to a wider range of plausible drivers was not our goal. Instead, our approach was to use the mixed model to generate effect sizes to allow a comparison of a limited set of important drivers.

Site was fitted as a random intercept with pH, shading, SOM, RelAm and year as fixed effects. In a small number of cases (five sites in year 1 and six sites in year 2) there were strong correlations between explanatory variables when examined within groups (Spearman correlation > ¦0.80¦). These sites were removed from the analysis, which reduced the between variable correlations to ¦0.26¦ (Fig. S2). As the response variable was count data, a Poisson distribution and log link was initially used. However, this resulted in an overdispersed model (Gelman & Hill, 2007; Bolker *et al*., 2009) and therefore a negative binomial model was used after confirming the lack of overdispersion. All possible combinations of variables of a global model were explored including interaction terms between (1) year and pH, shading, and RelAm and (2) shading and RelAm. The ‘dredge’ function of the MuMIn package (Barton, 2020) was used to extract the model with the lowest Akaike Information Criterion (AIC). We used the performance package (Lüdecke *et al*., 2021) to extract the conditional and marginal *R* ^2^ (Nakagawa & Schielzeth, 2013; Nakagawa *et al*., 2017) of the lowest AIC model. Regression coefficients were standardised and used to assess variable importance (Nakagawa & Cuthill, 2007; Gelman, 2008; Schielzeth, 2010). Significant variables were those whose regression parameters had 95% confidence intervals that did not include zero. We also considered the square of pH as plant species richness has been shown to have a unimodal response to soil pH in woodlands (Gould & Walker, 1999; Peppler‐Lisbach & Kleyer, 2009; and please refer to Fig. S3). Holly (*Ilex aquifolium*) and hawthorn (*Crataegus monogyna*) are two of the most common tree species found in British woodlands, and assignment of mycorrhizal type was considered weak for these plants. We therefore conducted a sensitivity analysis in which the mycorrhizal type was varied between AM and EM for hawthorn and AM and unknown for holly. In each instance the modelling process described above was repeated.

Spatial autocorrelation was tested by examining spline correlograms of the fitted model Pearson residuals (Bjørnstad & Falck, 2001; Zuur *et al*., 2009). The residuals showed no increase in spatial autocorrelation at short distances (Fig. S4).

## Results

### Effect of canopy mycorrhizal type on understory herbaceous species richness

**Fig. 1:**
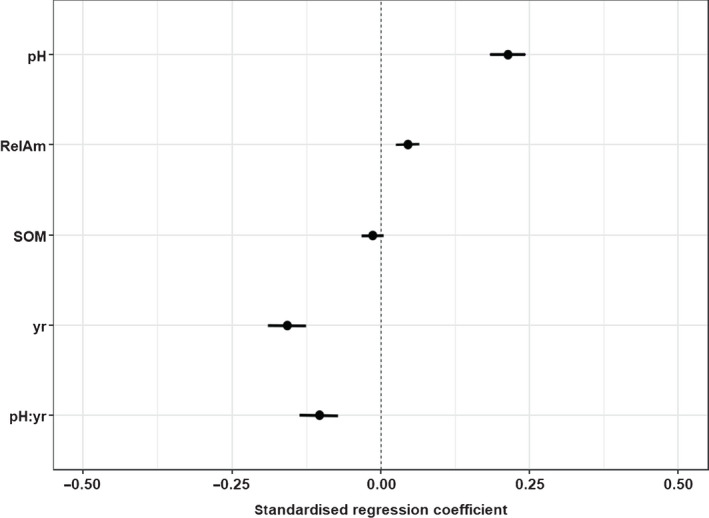
Effect of key explanatory factors on the understory richness of herbaceous plants using standardised regression coefficients with 95% confidence intervals. The explanatory variables were centred such that 1SD change in the variable results in the effect size change in the response (SD pH = 1.22, SD relative abundance of AM type trees and shrubs (RelAm) = 0.34). The relative abundance of arbuscular mycorrhizal (AM) trees and shrubs has a significant positive effect, as does soil pH. The effect of soil organic matter (SOM) is not significant. The effect of year, and the interaction between year and pH are negative. Conditional *R* ^2^ 0.492, marginal *R* ^2^ 0.114.

The AIC ‘best’ model (lowest AIC) of understory herb α‐diversity contained pH, RelAm, SOM, year and the interaction between year and pH (Fig. 1); details of all six models with ΔAIC ≤ 2 are provided in Table S2. The relative abundance of AM trees and shrubs (RelAm) had a significant positive effect on understory herb species richness, as did the soil pH, whereas the effect due to SOM was not significant. The effect of year, and the interaction between year and pH were both negative and significant. In the set of six candidate models (models within ΔAIC ≤ 2 of the AIC ‘best’ model) the same effects and interaction term were always significant, and neither SOM nor shading were statistically significant (Table S2). Using the transformation of pH to pH^2^ did not decrease AIC or increase *R* ^2^ in any model. The effect size for the random effect of site was larger than that of the explanatory variables (3.24 ± 0.042), indicating that unknown site‐specific factors explained variation in understorey richness in addition to the fixed effects. Sensitivity analyses revealed that the models were not sensitive to changes in the mycorrhizal status of *Ilex aquifolium* or *Crataegus monogyna*, and changes in the mycorrhizal type did not alter the variable set in the model with the lowest AIC (Table S3).

## Discussion

We asked whether the proportion of AM type trees and woody shrubs affected herbaceous plant species richness in British broadleaved woodlands. We found that, in agreement with the mycorrhizal mediation hypothesis (Veresoglou *et al*., 2017), the proportion of AM type trees and shrubs had a positive effect on herbaceous plant species richness. An important outcome of our approach was that it revealed the temporal consistency of this positive effect over the three decades between surveys. We were also able to show, for the first time, how the strength of the effect due to woody plant mycorrhizal type compared with other factors known to influence herbaceous plant species richness.

In this analysis we considered shading, SOM content and soil pH. Soil pH had the strongest effect, and a negative interaction with year. The positive effect of soil pH is seen because woodlands tend to have a lower pH, which is optimal for plant richness. In the Bunce woodlands, for example, the median soil pH is *c*. pH 4.75, whereas maximum plant richness is seen at between pH 5.5 and 6.0. Therefore, any increase in soil pH would correlate with an increase in plant richness. The negative interaction with year indicated that the positive effect of soil pH decreased across the 2 yr of the survey. This is probably due to increased shading in the woodlands. As woodland shading increases, the plant community shifts to more shade tolerant species. Therefore, any richness response to soil pH occurs within this limited community. This could have the effect of supressing herbaceous plants response to pH variability.

The significant negative effect of year on understory plant species richness was expected and reflects the general reduction in herbaceous richness seen in these woodlands between the 2 Bunce survey years. In our models, the interaction terms between the relative abundance of AM type trees and soil pH, year or shading, were either not contained in or were not significant in any models within ΔAIC ≤ 2, demonstrating that the RelAm effect was robust, despite the successional changes in these woodlands. In previous explorations of the mycorrhizal mediation hypothesis, a significant positive correlation between AM type woody plants and herbaceous species was found in mature ancient woodlands (Veresoglou *et al*., 2017). We found that the effect of the mycorrhizal type of the canopy was more important than shading, in both mature and less mature woodlands, suggesting that mycorrhizal mediation affects understory richness of both shaded and unshaded plant communities.

In our study we quantified the amount of AM type woody plants to link above ground plant abundance with belowground AM fungal inoculum potential, and thereby build on previous work to address the mycorrhizal mediation hypothesis (Veresoglou *et al*., 2017). However, if the abundance of woody plants does indeed imply greater abundance of AM fungi, it may in turn imply greater AM fungal richness, assuming richness is positively correlated with abundance. AM plant diversity has been shown to correlate with AM fungal diversity through differential resource acquisition (van der Heijden *et al*., 1998; Kernaghan, 2005). For example, grassland plant richness has been found to be positively correlated with AM fungal richness (Hiiesalu *et al*., 2014). We note that this effect is not consistent, other studies have found no relationship between aboveground plant richness and AM fungal diversity (Öpik *et al*., 2008) or found a significant relationship with plant diversity rather than plant richness (Mirzaei & Moradi, 2017). Alternatively, a negative relationship between plant richness and mycorrhiza formation has been found in temperate grasslands (Leon *et al*., 2022). Plant responses to AM fungi vary, so any plant diversity response may depend on soil conditions and AM fungal species identity (Vogelsang *et al*., 2006). We could not ascertain in this work whether the PSF mechanism driving understory richness was inoculum potential through AM fungal abundance or niche exploitation through AM fungal richness, therefore future work could examine empirical data on both AM fungal richness and inoculum potential and explore the correlations between AM woody plant cover and AM fungal richness. Interestingly, Mirzaei & Moradi (2017) measured spore density and found a significant relationship between AM fungal spore density and plant diversity, but not plant richness. In that work, plant richness was only significantly correlated with AM fungal colonisation, which could also be considered as a measure of inoculum potential.

In our analysis, the effect size for the random intercept was greater than that of any of the fixed effects, suggesting that historical legacies and local landscape scale effects are likely to have been important drivers of woodland plant species richness. The importance of these factors in British woodlands has been demonstrated by several authors. For example, Peterken & Game (1984) found that ancient woodlands in Lincolnshire, in the east of England, had greater understorey species richness, as did newer woods connected to ancient woodlands, whereas isolated newer woodlands were species poor. Woodland species tend to have poor dispersal characteristics (Kimberley *et al*., 2014), implying that, unless habitat connectivity is high, these species may fail to colonise new woodlands. Similarly, Petit *et al*. (2004) found that woodland plant species richness in England is correlated with woodland patch size; however, the authors also found that this effect did not persist for upland woods, where light and soil pH were more important. Other factors known to influence woodland plant richness include disturbance (Boch *et al*., 2013) or windthrow (Smart *et al*., 2014), nitrogen deposition, shading, habitat heterogeneity and land use around the woodland (Dzwonko & Loster, 1988; Petit *et al*., 2004; Brudvig *et al*., 2009). All these factors will increase the between‐site variance and contribute to the effect size of the random intercept.

The positive effect of the proportion of AM trees and shrubs on herb species richness supports previous findings (Newman & Reddell, 1988; Veresoglou *et al*., 2017) and further strengthens the case for the mycorrhizal mediation hypothesis by demonstrating this effect for the first time across over 100 British woodlands and 30 yr. The importance of identifying tree mycorrhizal type as a driver of understory species richness is that, unlike edaphic or climatic properties for example, it is a factor over which we can exert control in woodland management. If management plans depend on natural regeneration, then in a fragmented landscape and in woodlands dominated by a low diversity of EM type trees, AM type trees could be excluded with a negative effect on herbaceous plant species richness. This work suggests that the relatively straightforward practice of interplanting AM type hosts may be a tractable approach to increase woodland biodiversity. Or, when planning to plant new woodlands, the proportion of AM type and EM type hosts could be considered from the perspective of their influence on understory plant biodiversity.

We have shown that herb species richness is positively associated with the proportion of AM type trees and shrubs in British woodlands, and for the first time we show that this effect is robust across 30 yr of woodland succession. Our study builds on and expands previous work that has shown a link between overstorey mycorrhizal type and understorey species richness (Newman & Reddell, 1988; Veresoglou *et al*., 2017; Grünfeld *et al*., 2019). Finally, our results demonstrate that the effect due to AM type trees and shrubs is significant when compared with other important drivers of woodland plant species richness across a large‐scale national gradient of climate, soil and woodland type.

## Author contributions

PG, BJP and MT designed the project, PG carried out the modelling and wrote the manuscript with contributions from BJP, SMS and RS All authors reviewed and commented on the manuscript.

## Competing interests

None declared.

## Supporting information


**Fig. S1** Distribution of the mycorrhizal type of broadleaved tree species in woodlands in Great Britain.
**Fig. S2** Correlation plot.
**Fig. S3** Species richness response of understory in Bunce woodlands to soil pH.
**Fig. S4** Spline correlogram showing lack of spatial autocorrelation in the dataset.
**Methods S1** Brief description of the allocation of mycorrhizal type to the Bunce survey.
**Table S1** Mycorrhizal type of British trees and shrubs.
**Table S2** Details of the six models predicting understory species richness with ΔAIC < 2.
**Table S3** Sensitivity analyses.
Please note: Wiley Blackwell are not responsible for the content or functionality of any Supporting Information supplied by the authors. Any queries (other than missing material) should be directed to the *New Phytologist* Central Office.

## Data Availability

The Bunce survey is available from the UK Centre for Ecology and Hydrology https://catalogue.ceh.ac.uk/documents/ddff0f17‐c95d‐4415‐80cb‐aa9487edcb06.
