# Supplementary material for: Mycorrhizal type of woody plants influences understory species richness in British broadleaved woodlands
Source: New Phytol. 2022 Jun 15;235(5):2046–53. doi: 10.1111/nph.18274 (PMC9543792; doi:10.1111/nph.18274)
Supplement: Supplementary file 1 — Fig. S1 Distribution of the mycorrhizal type of broadleaved tree species in woodlands in Great Britain. Fig. S2 Correlation plot. Fig. S3 Species richness response of understory in Bunce woodlands to soil pH. Fig. S4 Spline correlogram showing lack of spatial autocorrelation in the dataset. Methods S1 Brief description of the allocation of mycorrhizal type to the Bunce survey. Table S1 Mycorrhizal type of British trees and shrubs. Table S2 Details of the six models predicting understory species richness with ΔAIC < 2. Table S3 Sensitivity analyses. Please note: Wiley Blackwell are not responsible for the content or functionality of any Supporting Information supplied by the authors. Any queries (other than missing material) should be directed to the New Phytologist Central Office. [file NPH-235-2046-s001.pdf]

New Phytologist **Supporting Information**

Mycorrhizal type of woody plants influences understory species richness in British broadleaved woodlands

Author list: Petra Guy, Richard Sibly, Simon M. Smart, Mark Tibbett, Brian J. Pickles

Article acceptance date: 12 May 2022

Methods S1. Brief description of the allocation of mycorrhizal type to the Bunce survey

Table S1. Summary of mycorrhizal type allocation for British woody plants.
Table S2. Effect sizes, standard errors, significance, AIC values, ΔAIC, and conditional and marginal R2 for the six lowest AIC models (all models with ΔAIC < 2).
Table S3. Sensitivity analyses

Figure S1. Distribution of the mycorrhizal type of broadleaved tree species in woodlands in Great Britain based on data from the National Forest Inventory (NFI) for 2011
Figure S2. Correlation plot for all pooled data across both years of the Bunce survey, after, sites with correlated variables were removed, with smoothed regression lines. Upper
Figure S3. Species richness response of understory in Bunce woodlands to soil pH
Figure S4. Spline correlogram with 95% confidence intervals of the Pearson residuals of the mixed effects negative binomial model.

Methods S1. Allocation of mycorrhizal type to trees and woody shrubs in the Bunce survey.

Mycorrhizal type is assigned based on structures formed by the colonizing fungus within plant roots. Ectomycorrhizal fungi form a Hartig net within the root cortical cells and a sheath around the root tip. Root changes or infections of non-mycorrhizal fungi which give the appearance of a sheath or Hartig net could lead the plant being erroneously interpreted as EM type (Brundrett & Tedersoo 2020; Tedersoo & Brundrett 2017).

Definition of AM type is a developing field (Brundrett & Tedersoo 2019, Bueno *et al.,* 2019a, Bueno *et al.,* 2019b; Bueno *et al.,* 2021), but infection by AMF fungi can result in the presence of arbuscules (the generally accepted definition of AM type), vesicles, hyphae, or all of these structures. This means that plants defined as AM type in literature may fit a range of criteria.

Errors in the assignment of type may also occur simply due to lack of data. Some plants show low or fluctuating AMF colonisation rates. Therefore, some research may show AMF colonisation, whilst other work may not. In which case, a larger quantity of empirical data may be required in order to have confidence regarding the mycorrhizal type. A typical example of this was *Buddleja davidii* (Dickie *et al.,* 2007). One piece of data was cited in Harley and Harley (1987) in which no AMF colonisation was found, however, later work demonstrated that the plant could in fact be colonised by AMF.

Errors in the assignment of mycorrhizal type and the subsequent impact on analysis have been discussed in detail elsewhere (Brundrett and Tedersoo, 2019, 2020; Bueno *et al.,* 2019a; Tedersoo *et al.,* 2019) and attention has been brought to the uncritical use of mycorrhizal type databases since errors can be perpetuated. For this analysis we therefore conducted an in-depth review of mycorrhizal type of all woody plants in the Bunce data set using recent large databases as a starting point (Akhmetzhanova *et al.,* 2012; Soudzilovskaia *et al.*, 2020). We scrutinised each reference cited and sought additional work where data was scarce. We focussed on field work rather than glasshouse experiments since there is evidence that putatively EM trees will associate with AM fungi when stressed, or when EM fungal inoculum is in short supply (Cázares & Trappe, 1993, Cázares & Smith, 1995). We did not rely on the older databases of Harley and Harley (1987) or Wang & Qiu (2006) because both are now somewhat outdated due to more recent research, and the latter of these tends to rely heavily on the former for UK plants.

Due to the scale of the task, full details and discussion of the assignment of mycorrhizal type to British woody plants has been prepared as a stand-alone publication (P. Guy *et al.*, unpublished data).

Table S1. Summary of mycorrhizal type allocation for British woody plants. Column 3 shows mycorrhizal type assigned in this work: AM = arbuscular mycorrhizal fungal host, EM = ectomycorrhizal fungal host, Ar = arbutoid, Dual = plants which can host both arbuscular and ectomycorrhizal fungi, Er = ericoid mycorrhizal fungal host plant, U = unknown mycorrhizal type, insufficient data available to make an allocation.

**Species**	**Family**	**Type**
Acaena novae-zelandiae	Rosaceae	AM
Acer campestre	Sapindaceae	AM
Acer platanoides	Sapindaceae	AM
Acer pseudoplatanus	Sapindaceae	AM
Aesculus hippocastanum	Sapindaceae	AM
Alnus glutinosa	Betulaceae	Dual
Alnus incana	Betulaceae	Dual
Andromeda polifolia	Ericaceae	Er
Arctostaphylos alpinus	Ericaceae	Ar
Arctostaphylos uva-ursi	Ericaceae	Ar
Berberis vulgaris	Berberidaceae	AM
Betula nana	Betulaceae	EM
Betula pendula	Betulaceae	EM
Betula pubescens	Betulaceae	EM
Buddleja davidii	Scrophulariaceae	AM
Buxus sempervirens	Buxaceae	U
Calluna vulgaris	Ericaceae	Er
Carpinus betulus	Betulaceae	EM
Castanea sativa	Fagaceae	EM
Chamaecyparis lawsoniana	Cupressaceae	AM
Clematis vitalba	Ranunculaceae	AM
Colutea arborescens	Fabaceae	U
Cornus sanguinea	Cornaceae	AM
Cornus sericea	Cornaceae	AM
Corylus avellana	Betulaceae	EM
Cotoneaster spp	Rosaceae	AM
Crataegus laevigata	Rosaceae	AM
Crataegus monogyna	Rosaceae	AM
Cytisus scoparius	Fabaceae	AM
Daphne laureola	Thymelaeaceae	AM
Daphne mezereum	Thymelaeaceae	AM
Dryas octopetala	Rosaceae	EM
Empetrum nigrum	Ericaceae	Er
Erica ciliaris	Ericaceae	Er
Erica cinerea	Ericaceae	Er
Erica tetralix	Ericaceae	Er
Erica vagans	Ericaceae	Er
Euonymus europaeus	Celastraceae	AM
Fagus sylvatica	Fagaceae	EM
Frangula alnus	Rhamnaceae	AM

Fraxinus excelsior	Oleaceae	AM
Fuchsia magellanica	Onoagraceae	AM
Gaultheria shallon	Ericaceae	Er
Genista anglica	Fabaceae	AM
Genista pilosa	Fabaceae	AM
Genista tinctoria	Fabaceae	AM
Hedera helix	Araliaceae	AM
Hippophae rhamnoides	Elaeagnaceae	AM
Hypericum androsaemum	Hypericaceae	AM
Hypericum calycinum	Hypericaceae	AM
Ilex aquifolium	Aquifoliaceae	AM
Juglans regia	Juglandaceae	AM
Juniperus communis	Cupressaceae	AM
Laburnum anagyroides	Fabaceae	U
Larix decidua	Pinaceae	EM
Larix kaempferi	Pinaceae	EM
Laurus nobilis	Lauraceae	AM
Lavatera arborea	Malvaceae	AM
Leycesteria formosa	Caprifoliaceae	U
Ligustrum ovalifolium	Oleaceae	AM
Ligustrum vulgare	Oleaceae	AM
Loiseleuria procumbens	Ericaceae	Er
Lonicera periclymenum	Caprifoliaceae	AM
Lonicera xylosteum	Caprifoliaceae	AM
Lupinus arboreus	Fabaceae	NM
Mahonia aquifolium	Berberidaceae	AM
Malus domestica	Rosaceae	AM
Malus sylvestris	Rosaceae	AM
Mespilus germanica	Rosaceae	AM
Phyllodoce caerulea	Ericaceae	Er
Picea abies	Pinaceae	EM
Picea sitchensis	Pinaceae	EM
Pinus contorta	Pinaceae	EM
Pinus nigra	Pinaceae	EM
Pinus sylvestris	Pinaceae	EM
Populus alba	Salicaceae	Dual
Populus alba x tremula (P. x canescens)	Salicaceae	Dual
Populus nigra	Salicaceae	Dual
Populus tremula	Salicaceae	Dual
Potentilla fruticosa	Rosaceae	AM
Prunus spp	Rosaceae	AM
Pseudotsuga menziesii	Pinaceae	EM
Pyrus communis	Rosaceae	AM
Pyrus cordata	Rosaceae	AM
Quercus cerris	Fagaceae	EM
Quercus ilex	Fagaceae	EM

Quercus petraea	Fagaceae	EM
Quercus robur	Fagaceae	EM
Rhamnus cathartica	Rhamnaceae	AM
Rhododendron ponticum	Ericaceae	Er
Ribes alpinum	Grossulariaceae	AM
Ribes nigrum	Grossulariaceae	AM
Ribes rubrum	Grossulariaceae	AM
Ribes spicatum	Grossulariaceae	AM
Ribes uva-crispa	Grossulariaceae	AM
Robinia pseudoacacia	Fabaceae	AM
Rosa spp	Rosaceae	AM
Rubus caesius	Rosaceae	AM
Rubus fruticosus agg.	Rosaceae	AM
Rubus idaeus	Rosaceae	AM
Rubus spectabilis	Rosaceae	AM
Ruscus aculeatus	Asparagaceae	AM
Salix alba	Salicaceae	EM
Salix arbuscula	Salicaceae	U
Salix aurita	Salicaceae	EM
Salix caprea	Salicaceae	EM
Salix cinerea	Salicaceae	EM
Salix fragilis	Salicaceae	EM
Salix herbacea	Salicaceae	EM
Salix lanata	Salicaceae	EM
Salix lapponum	Salicaceae	EM
Salix myrsinifolia	Salicaceae	U
Salix myrsinites	Salicaceae	EM
Salix pentandra	Salicaceae	EM
Salix phylicifolia	Salicaceae	EM
Salix purpurea	Salicaceae	AM
Salix repens	Salicaceae	Dual
Salix reticulata	Salicaceae	EM
Salix triandra	Salicaceae	EM
Salix viminalis	Salicaceae	EM
Sambucus nigra	Adoxaceae	AM
Sambucus racemosa	Adoxaceae	AM
Sorbus spp	Rosaceae	AM
Symphoricarpos albus	Caprifoliaceae	AM
Syringa vulgaris	Oleaceae	AM
Taxus baccata	Taxaceae	AM
Thuja plicata	Cupressaceae	AM
Tilia cordata	Malvaceae	EM
Tilia cordata x platyphyllos (T. x europaea)	Malvaceae	AM
Tilia platyphyllos	Malvaceae	EM
Tsuga heterophylla	Pinaceae	EM

Ulex europaeus	Fabaceae	AM
Ulex gallii	Fabaceae	AM
Ulex minor	Fabaceae	AM
Ulmus glabra	Ulmaceae	AM
Ulmus minor	Fabaceae	AM
Ulmus plotii	Ulmaceae	AM
Ulmus procera	Ulmaceae	AM
Vaccinium myrtillus	Ericaceae	Er
Vaccinium uliginosum	Ericaceae	Er
Vaccinium vitis-idaea	Ericaceae	Er
Viburnum lantana	Adoxaceae	AM
Viburnum opulus	Adoxaceae	AM

Table S2. Effect sizes, standard errors, significance, AIC values, ΔAIC, and conditional and marginal R^2 for the six lowest AIC models (all models with ΔAIC ≤ 2). The same set of variables (pH, RelAm, year, and the interaction between year and pH), highlighted in bold, are the only significant predictors of understory species richness in all models in this set. Asterisks indicate significance level. (pH; soil pH; RelAm, the relative abundance of AM trees and shrubs; SOM, soil organic matter content; yr, year; shading, calculated as the sum over the DBH classes multiplied by the number of stems in that class)

variable	effect size	std error	z value	Pr(>|z|)		AIC	ΔAIC	conditional R^2	marginal R^2
**ph**	0.31	0.03	11.13	< 2e-16	***	22262.07	0.00	0.49	0.11
**RelAm**	0.05	0.01	4.76	1.97e-06	***				
**SOM**	-0.01	0.01	-1.42	0.156					
**yr**	-0.16	0.02	-9.74	< 2e-16	***				
**ph:yr**	-0.10	0.02	-6.40	1.58e-10	***				
**ph**	0.31	0.03	11.11	< 2e-16	***	22262.09	0.02	0.49	0.11
**RelAm**	0.05	0.01	4.77	1.87e-06	***				
**yr**	-0.16	0.02	-9.80	< 2e-16	***				
**ph:yr**	-0.10	0.02	-6.34	2.28e-10	***				
**ph**	0.31	0.03	10.56	< 2e-16	***	22262.78	0.71	0.49	0.11
**RelAm**	0.07	0.03	2.80	0.00504	**				
**SOM**	-0.01	0.01	-1.43	0.1528					
**yr**	-0.16	0.02	-9.72	< 2e-16	***				
**ph:yr**	-0.10	0.02	-5.90	3.64e-09	***				
RelAm:yr	-0.02	0.02	-1.14	0.25533					
**ph**	0.31	0.03	10.55	< 2e-16	***	2262.82	0.75	0.49	0.11
**RelAm**	0.07	0.03	2.80	0.00519	**				
**yr**	-0.16	0.02	-9.79	< 2e-16	***				
**ph:yr**	-0.10	0.02	-5.85	4.98e-09	***				
RelAm:yr	-0.02	0.02	-1.12	0.26131					
**ph**	0.31	0.03	10.52	< 2e-16	***	22263.42	1.35	0.49	0.11
**RelAm**	0.08	0.03	2.95	0.00322	**				
**yr**	-0.16	0.02	-9.42	< 2e-16	***				
**shading**	0.00	0.01	0.18	0.85808					
**ph:yr**	-0.10	0.02	-5.83	5.55e-09	***				
**RelAm:shading**	-0.02	0.01	-1.77	0.07723	.				
**RelAm:yr**	-0.02	0.02	-1.44	0.14917					
**ph**	0.32	0.03	11.09	< 2e-16	***	22263.51	1.44	0.49	0.11
**RelAm**	0.04	0.01	4.28	1.90e-05	***				
**yr**	-0.16	0.02	-9.44	< 2e-16	***				
**shading**	0.00	0.01	0.17	0.866					
**ph:yr**	-0.10	0.02	-6.37	1.95e-10	***				

**RelAm:shading**	-0.02	0.01	-1.53	0.125					

Table S3. Sensitivity analysis. Allocation of mycorrhizal type to *Crataegus monogyna* and *Ilex aquifolium* was questionable. Although it is likely that *C. monogyna* is AM type in line with other members of the Rosaceae family, there was some suggestion in the literature that this plant might associate with EMF in woodland settings. *I. aquifolium* is likely to be AM type in line with other *Ilex* spp., however, in our literature search, we found no work looking at this specific species. Therefore, we carried out sensitivity analysis by changing the mycorrhizal type of these two plants. In the main model both *C. monogyna* and *I. aquifolium* are allocated as AM type. The model was repeated with three different permutations: *C. mongyna* AM, *I. aquifolium* unknown; *C monogyna* EM, *I. aquifolium* AM; *C. monogyna* EM, *I. aquifolium* unknown. In each case, the same full global model was created and the ‘dredge’ function was used to extract the model with the lowest AIC. The table shows effect sizes for the lowest AIC model when the mycorrhizal status of C. *monogyna* and *I. aquifolium* were changed. The results are not sensitive to changes in the mycorrhizal type of these plants; the same set of variables appear in the lowest AIC model in every permutation. Asterisks indicate significance level. (pH; soil pH; RelAm, the relative abundance of AM trees and shrubs; SOM, soil organic matter content; yr, year; shading, calculated as the sum over the DBH classes multiplied by the number of stems in that class)

**variable**	**effect size**	**std error**	**z value**	**Pr(>|z|)**		**AIC**	**conditional R^2**	**marginal R^2**
Crataegus: AM. *Ilex*: unknown								
**ph**	0.31	0.03	11.02	< 2e-16	***	22246.01	0.50	0.12
**RelAm**	0.06	0.01	6.23	4.76e-10	***			
**SOM**	-0.01	0.01	-1.44	0.15				
**yr**	-0.15	0.02	-9.59	< 2e-16	***			
**ph:yr**	-0.10	0.02	-6.43	1.32e-10	***			
Crataegus: EM. *Ilex*: AM								
**ph**	0.32	0.03	11.18	< 2e-16	***	22261.46	0.49	0.11
**RelAm**	0.05	0.01	4.82	1.43e-06	***			
**SOM**	-0.01	0.01	-1.46	0.144				
**yr**	-0.16	0.02	-9.76	< 2e-16	***			
**ph:yr**	-0.10	0.02	-6.43	1.29e-10	***			
Crataegus: EM. *Ilex*: unknown								
**ph**	0.31	0.03	11.09	< 2e-16	***	22244.68	0.50	0.12
**RelAm**	0.06	0.01	6.33	2.42e-10	***			
**SOM**	-0.01	0.01	-1.49	0.135				
**yr**	-0.15	0.02	-9.63	< 2e-16	***			
**ph:yr**	-0.10	0.02	-6.47	1.00e-10	***			

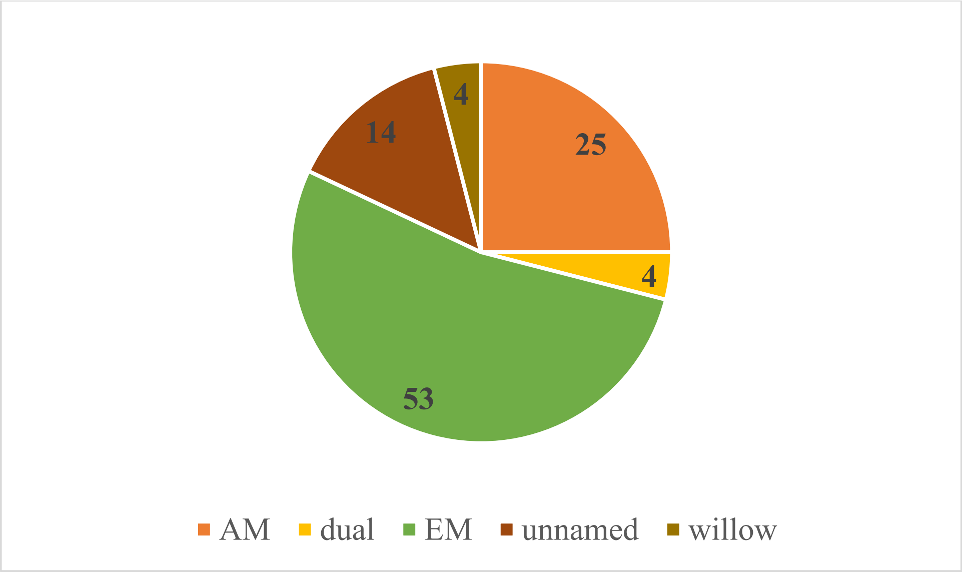

Figure S1. Distribution of the mycorrhizal type of broadleaved tree species in woodlands in Great Britain based on data from the National Forest Inventory (NFI) for 2011 (National Forest Inventory, 2012). (AM, arbuscular mycorrhizal. EM, ectomycorrhizal, dual, thought to host both arbuscular and ectomycorrhizal fungi. Unnamed, these trees were not identified in the NFI and hence mycorrhizal type could not be allocated). EM trees specified in the NFI are oak (*Quercus robur, Q. petraea)*, beech, (*Fagus sylvatica),* birch (*Betula pendula, B. pubescens)*, sweet chestnut (*Castanea sativa*, and hazel (*Corylus avellana)*. Birch is sometimes referred to as a dual host, but we found little evidence for this in British woodlands. AM trees are sycamore *(Acer pseudoplatanus)*, hawthorn (*Crataegus monogyna),* and ash (*Fraxinus excelsior)*. Dual mycorrhizal trees are alder (*Alnus glutinosa)*. Willows (*Salix spp.)* are shown separately as they can be either EM or AM type. Note that even if all unnamed species were AM type hosts (e.g., rowan (*Sorbus* spp.) or holly (*Ilex aquifolium*)), the proportion of EM type hosts in British woodlands is still greater.

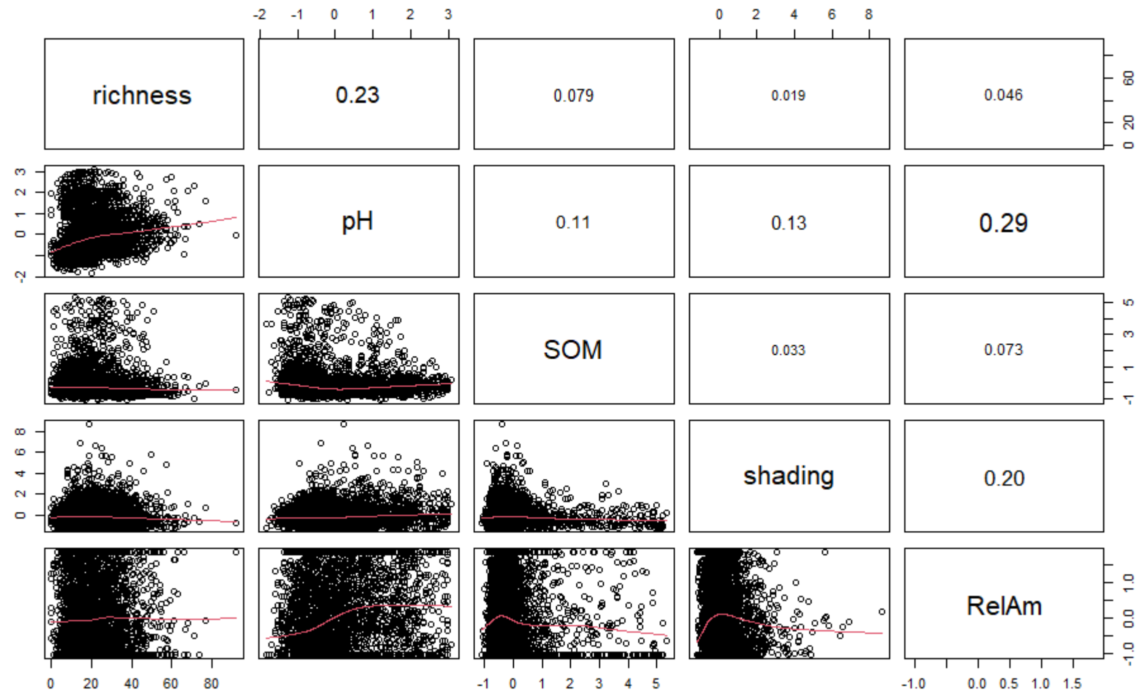

Figure S2. Correlation plot for all pooled data across both years of the Bunce survey, after, sites with correlated variables were removed, with smoothed regression lines. Upper panel shows Spearman correlation coefficient. (Richness, understory herb richness; pH, soil pH; SOM, soil organic matter; shading; shading, calculated as the sum over the DBH classes multiplied by the number of stems in that class; RelAm, relative abundance of AM type trees and shrubs)

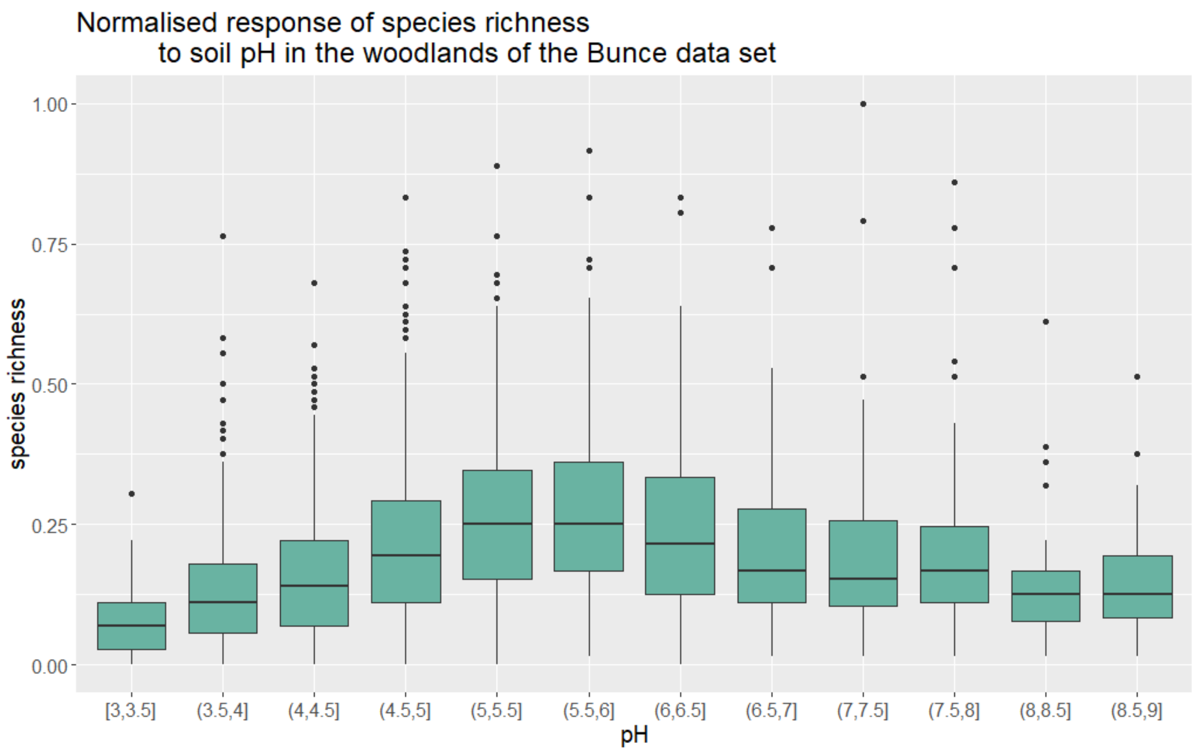

Figure S3. Response of understory plant species richness in woodlands of the Bunce dataset to soil pH. The soil pH has been grouped into bin widths of 0.5. The peak richness can be seen at around pH 5.5 to pH 6. Horizontal lines on the box-plots indicate the median, the 1st and 3rd quartiles. Whiskers denote 1.5x the inter quartile range (IQR) and points denote values outside the IQR. Square and round brackets denote the half open interval (a, b] = a <x ≤b.

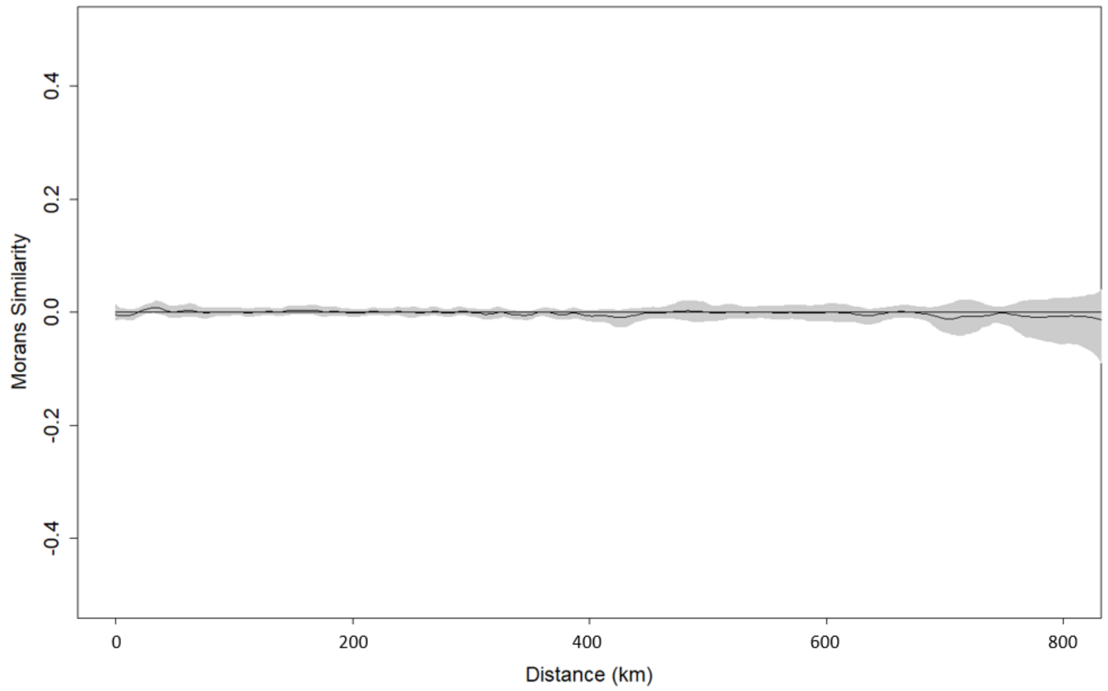

Figure S4. Spline correlogram with 95% confidence intervals of the Pearson residuals of the mixed effects negative binomial model. There is no evidence of significant spatial autocorrelation, which suggests that i) the mixed effects model takes account of any within site spatial autocorrelation, and ii) between site autocorrelation is negligible.

References

Akhmetzhanova, AA., Soudzilovskaia, NA, Onipchenko, VG, Cornwell, WK, Agafonov, VA, Selivanov, IA, & Cornelissen, JHC. 2012. A rediscovered treasure: mycorrhizal intensity database for 3000 vascular plant species across the former Soviet Union. Ecology, 93(3), 689–690.

Brundrett, MC. 2009. Mycorrhizal associations and other means of nutrition of vascular plants: Understanding the global diversity of host plants by resolving conflicting information and developing reliable means of diagnosis. *Plant and Soil*, *320*(1–2), 37–77.

Brundrett MC, Tedersoo L. 2020. Resolving the mycorrhizal status of important northern hemisphere trees. *Plant and Soil* 454: 3–34.

Brundrett MC. 2021. Auditing data resolves systemic errors in databases and confirms mycorrhizal trait consistency for most genera and families of flowering plants. *Mycorrhiza* 31 671-683

Brundrett MC, Tedersoo L. 2019. Misdiagnosis of mycorrhizas and inappropriate recycling of data can lead to false conclusions. *New Phytologist* 221: 18–24.

Bueno CG, Aldrich-Wolfe L, Chaudhary VB, Gerz M, Helgason T, Hoeksema JD, Klironomos J, Lekberg Y, Leon D, Maherali H, 2019a. Misdiagnosis and uncritical use of plant mycorrhizal data are not the only elephants in the room. *New Phytologist* 224: 1415–1418.

Bueno CG, Gerz M, Zobel M, Moora M. 2019b. Conceptual differences lead to divergent trait estimates in empirical and taxonomic approaches to plant mycorrhizal trait assignment. *Mycorrhiza* 29: 1-11.

Bueno CG, Davison J, Leon D, Meng Y, Öpik M, Zobel M, Moora M. 2021. Towards a consistent benchmark for plant mycorrhizal association databases. *New Phytologist* 231: 913–916.

Cázares, E, & Trappe, JM. 1993. Vesicular endophytes in roots of the Pinaceae. *Mycorrhiza*, 2(4), 153–156.

Cázares, E, & Smith, JE. 1995. Occurrence of vesicular-arbuscular mycorrhizae in Pseudotsuga menziesii and *Tsuga heterophylla* seedlings grown in Oregon Coast Range soils. *Mycorrhiza*, 6(1), 65–67.

Dickie, IA, Thomas MM, & Bellingham PJ. 2007. On the perils of mycorrhizal status lists: The case of *Buddleja davidii. Mycorrhiza*, 17(8), 687–688

Harley Jl, Harley EL. 1987. A Check-List of Mycorrhiza in the British Flora. New Phytologist, 105(2), 1–102.

Hartnett DC, Wilson GWT. 2002. The role of mycorrhizas in plant community structure and dynamics: Lessons from grasslands. *Plant and Soil* 244: 319–331.

National Forest Inventory. 2012. NFI preliminary estimates of quantities of broadleaved species in British woodlands, with special focus on ash. Forestry Commission: Edinburgh, UK.

Soudzilovskaia NA, Vaessen S, Barcelo M, He J, Rahimlou S, Abarenkov K, Brundrett MC, Gomes SI, Merckx V, Tedersoo L. 2020. FungalRoot: Global online database of plant mycorrhizal associations. *New Phytologist* 227: 955-966.

Wang B, Qiu YL. 2006. Phylogenetic distribution and evolution of mycorrhizas in land plants. *Mycorrhiza*, 16(5), 299–363.

Tedersoo L, Rahimlou S, Brundrett MC. 2019. Misallocation of mycorrhizal traits leads to misleading results. *Proceedings of the National Academy of Sciences of the United States of America* 116: 12139–12140.
